# Application research of radiomics in colorectal cancer: A bibliometric study

**DOI:** 10.1097/MD.0000000000037827

**Published:** 2024-04-12

**Authors:** Lihong Yang, Binjie Wang, Xiaoying Shi, Bairu Li, Jiaqiang Xie, Changfu Wang

**Affiliations:** a Department of Radiology and Medical Imaging Research Institute, Huaihe Hospital of Henan University, Kaifeng, Henan, China; b Department of Breast and Thyroid Surgery, Huaihe Hospital of Henan University, Kaifeng, Henan, China.

**Keywords:** bibliometrics, colorectal cancer, radiomics

## Abstract

**Background:**

Radiomics has shown great potential in the clinical field of colorectal cancer (CRC). However, few bibliometric studies have systematically analyzed existing research in this field. The purpose of this study is to understand the current research status and future development directions of CRC.

**Methods:**

Search the English documents on the application of radiomics in the field of CRC research included in the Web of Science Core Collection from its establishment to October 2023. VOSviewer and CiteSpace software were used to conduct bibliometric and visual analysis of online publications related to countries/regions, authors, journals, references, and keywords in this field.

**Results:**

A total of 735 relevant documents published from Web of Science Core Collection to October 2023 were retrieved, and a total of 419 documents were obtained based on the screening criteria, including 376 articles and 43 reviews. The number of publications is increasing year by year. Among them, China publishes the most relevant documents (n = 238), which is much higher than Italy (n = 69) and the United States (n = 63). Tian Jie is the author with the most publications and citations (n = 17, citations = 2128), GE Healthcare is the most productive institution (n = 26), *Frontiers in Oncology* is the journal with the most publications (n = 60), and *European Radiology* is the most cited journal (n = 776). Hot spots for the application of radiomics in CRC include magnetic resonance, neoadjuvant chemoradiotherapy, survival, texture analysis, and machine learning. These directions are the current hot spots for the application of radiomics research in CRC and may be the direction of continued development in the future.

**Conclusion:**

Through bibliometric analysis, the application of radiomics in CRC has been increasing year by year. The application of radiomics improves the accuracy of preoperative diagnosis, prediction, and prognosis of CRC. The results of bibliometrics analysis provide a valuable reference for the research direction of radiomics. However, radiomics still faces many challenges in the future, such as the single nature of the data source which may affect the comprehensiveness of the results. Future studies can further expand the data sources and build a multicenter public database to more comprehensively reflect the research status and development trend of CRC radiomics.

## 1. Introduction

Colorectal cancer (CRC) is the third most common malignant tumor in the world and the second leading cause of death from malignant tumors. According to the latest global cancer burden data released by the World Health Organization’s International Agency for Research on Cancer, there were 1.93 million new cases of CRC in 2020, leading to About 1.07 million deaths.^[1]^ Approximately 56% of CRC patients are in the intermediate or advanced stage when they are diagnosed.^[2]^ Patients with early-stage CRC have a 90% survival rate, while the survival rate for intermediate- and late-stage CRC is only 20%.^[3]^ Radiomics is a high-throughput tool that extracts a large number of image features and converts them into high-dimensional data. Due to the explosive growth of imaging and clinical data, as well as breakthrough research in artificial intelligence, radiomics has important applications in CRC clinical diagnosis,^[4,5]^ tumor prediction,^[6,7]^ and treatment decisions.^[8,9]^ With the widespread use of radiomics technology, a large number of high-quality documents related to the application of CRC radiomics have emerged. Despite the numerous advancements made in CRC radiomics, there are still unresolved issues and controversial points. For instance, the standardization and reproducibility of radiomics methods remain challenges. Different research teams may adopt distinct algorithms and parameter settings, leading to inconsistencies in results. This study used VOSviewer^[10]^ and CiteSpace software to conduct a comprehensive bibliometric analysis of the documents related to CRC radiomics research in the WoSCC from its inception to October 2023. By summarizing the past research findings, we aim to provide a comprehensive and profound understanding of the current application status and future development trends of radiomics in CRC research, to promote the sustained development of radiomics in the medical field.

## 2. Methods

### 2.1. Data sources and search strategies

The statistical analysis data of this article comes from WoSCC, search formula (TS=“Colorectal cancer” OR “Colon cancer” OR “Rectal cancer” OR “CRC” OR “LARC” OR “LACC”) AND (TS=” Radiomics” OR “imageomics”), the language type is limited to “English,” the document type is limited to articles and reviews, the period is from the establishment of the database to October 31, 2023. To ensure the quality of the screened documents, 2 researchers screened the documents separately based on their content. Documents irrelevant to the topics “CRC” and “Radiomics” were excluded, and 419 documents were finally retained after verification, comparison, and deduplication. Export in the format of “tab-delimited file” and select “Full record and cited references” for the record content. The final exported data includes the title, keywords, author, institution, address, abstract, and publication time of each document.

### 2.2. Research methods

Bibliometric analysis was carried out on the above screened, checked, and deduplicated literature. Origin 2022 software was used to draw the annual publication volume chart, and VOSviewer and Citespace software was used to conduct bibliometric and visual analysis of online publications related to countries/regions, authors, journals, references, and keywords. The VOSviewer software assisted us in analyzing the collaborative relationships and influence among countries/regions, authors, and journals in this field, while the Citespace software was used to analyze the co-occurrence and evolution trends of references and keywords. The use of these tools enabled us to gain a more comprehensive understanding of the current research status and development trends in the field of CRC radiomics.

## 3. Results

### 3.1. General results

**Figure 1.**
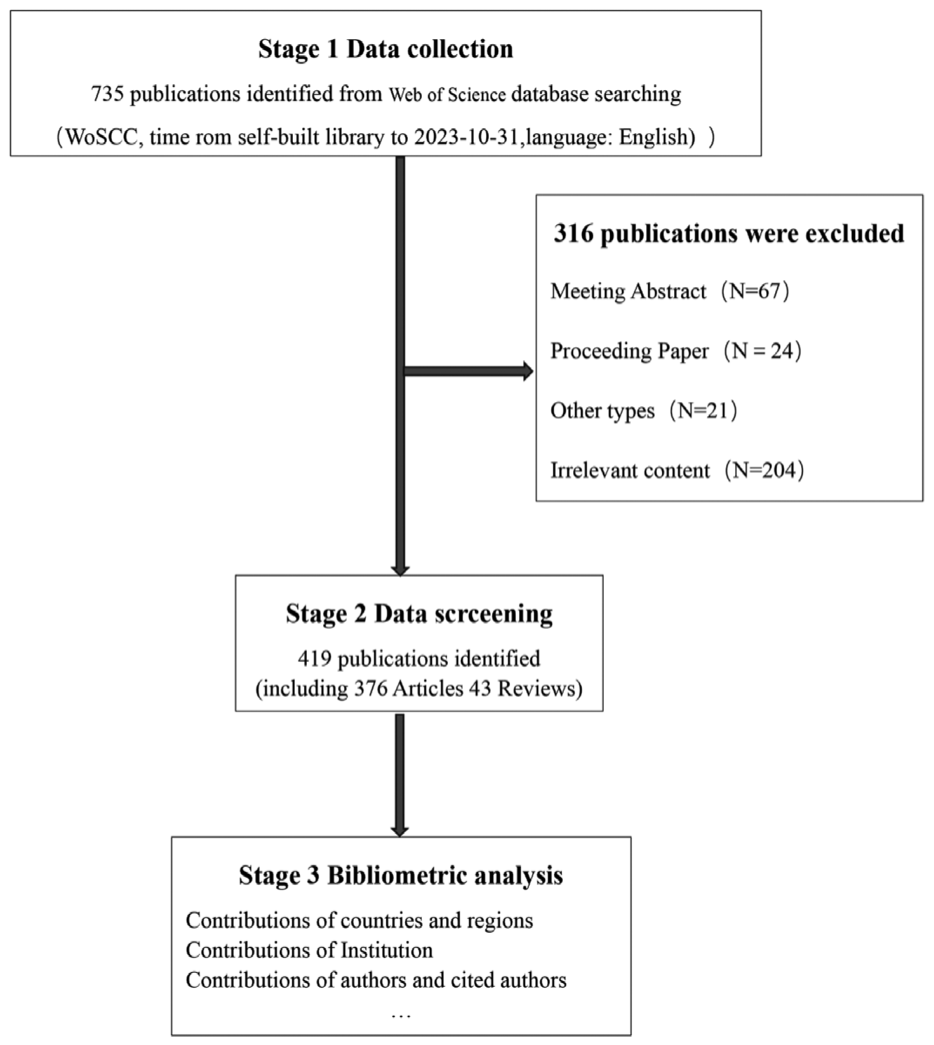
Detailed flowchart of search. WoSCC = Web of Science Core Collection.

The initial review yielded 735 relevant documents. According to the inclusion and exclusion criteria (Fig. 1), 419 relevant documents on the application of radiomics in CRC were finally included, of which 376 (51.1%) were articles and 43 (5.8%) were reviewed. Three hundred seventeen documents (43.1%) were excluded from conference abstracts, proceedings documents, and other documents irrelevant to the search content.

### 3.2. Analysis of the publication trend of radiomics applied to CRC research

**Figure 2.**
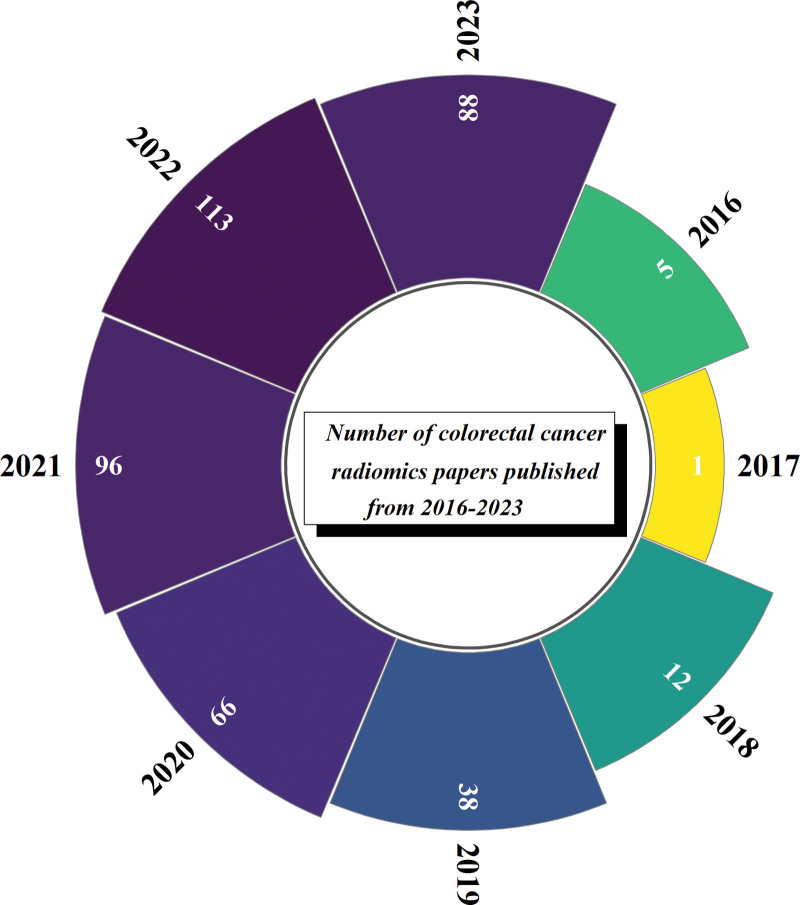
Trend chart of annual publication volume of clinical applications of radiomics in CRC from 2016 to 2023. CRC = colorectal cancer.

Statistics on the annual publication volume of WoSCC since the establishment of the database to October 31, 2023, found that the first research document on the application of radiomics in CRC appeared in 2016. From 2016 to 2018, the number of published documents increased from 5 to 12; from 2019 to 2022, the number of published documents surged from 38 to 113; from January 2023 to October 2023, the number of published documents reached 88 (Fig. 2).

### 3.3. Analysis of countries publishing publications on the application of radiomics in CRC research

**Figure 3.**
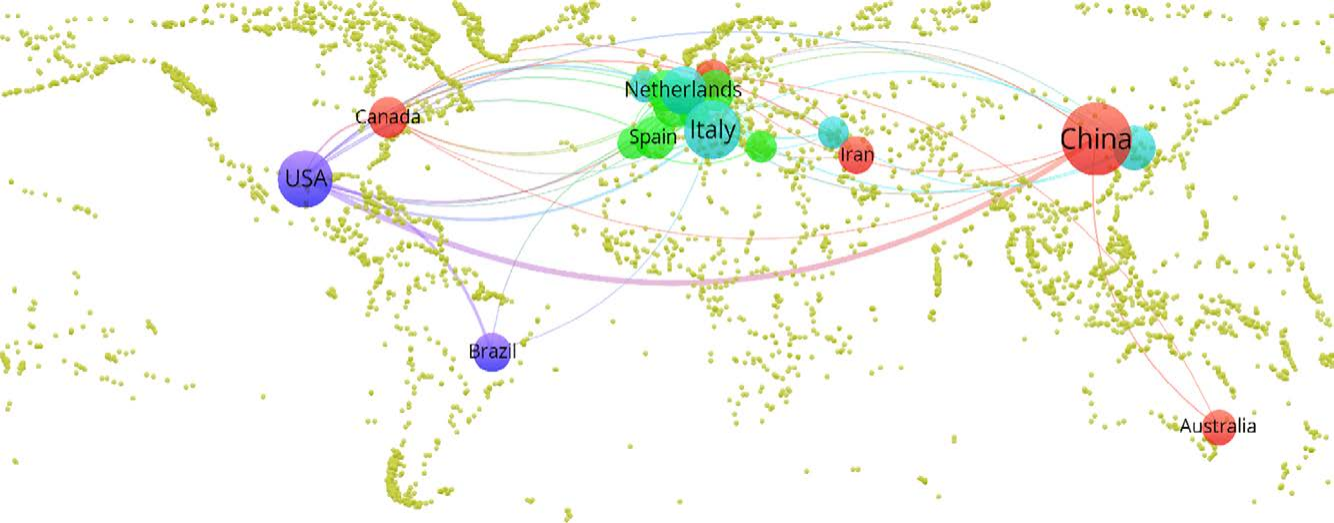
National (regional) cooperation network map of radiomics applied to CRC research. CRC = colorectal cancer.

**Figure 4.**
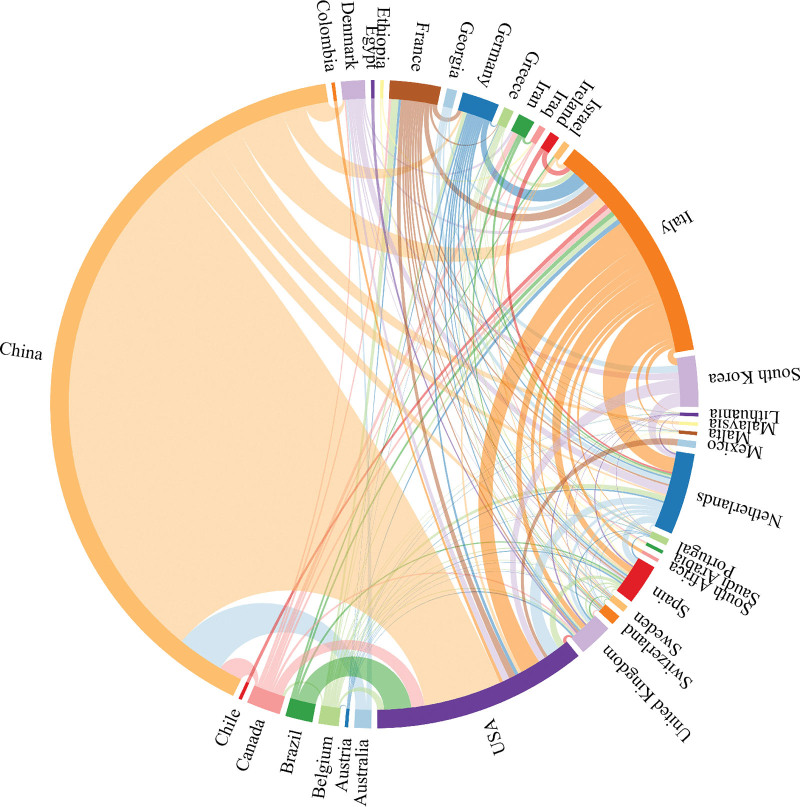
A visual diagram of cooperation between countries (regions). China and the United States publish a large amount of documents and have many cooperation contacts with other countries.

Use VOSviewer software to draw a cooperation network map of countries (regions) (Figs. 3–4). The nodes represent countries (regions), the size of the nodes represents the number of publications by countries (regions), and the lines represent the cooperative relationships between countries (regions). It can be seen that China, Italy, and the United States have larger nodes, and the number of published documents is more than 30. Among them, China has the largest number of published documents, with 238 documents. The total connection strength is greater among the United States, Italy, the Netherlands, and China, with the United States having the most connections at 57, indicating that the United States cooperates most closely with other countries.

### 3.4. Author analysis of publications on the application of radiomics in CRC research

**Figure 5.**
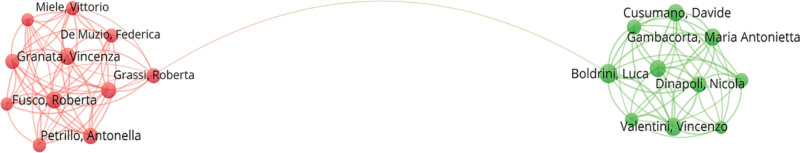
Author collaboration network map of radiomics applied to CRC clinical research. CRC = colorectal cancer.

**Figure 6.**
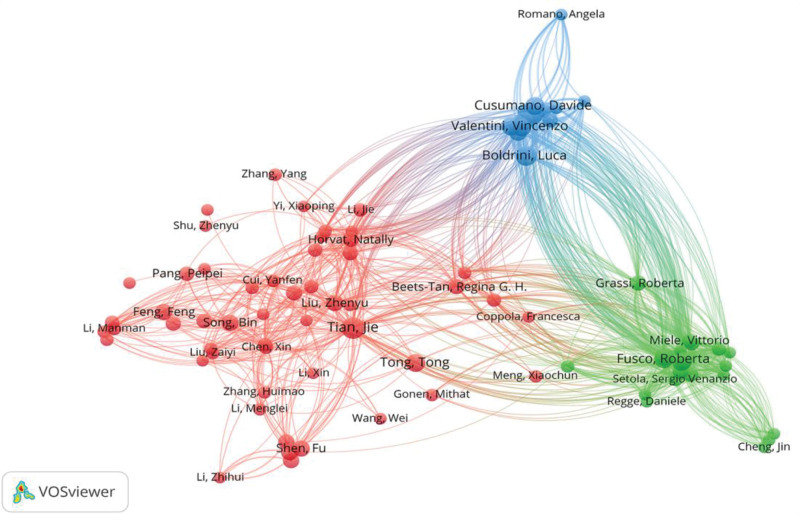
Cited author collaboration network map of radiomics applied to CRC clinical research. CRC = colorectal cancer.

The author’s collaboration network map reflects the collaborative relationships among researchers in the field of radiomics applied to CRC. In VOSviewer, select authors who have published more than 7 documents and draw a network map of author collaboration and co-occurrence (Fig. 5). The number of nodes in the network is 45, and the total number of connections is 1758. The nodes represent the number of documents published by the author. The larger the node, the more documents the author has published. The connections represent the cooperative relationship between the authors. The more connections, the closer the cooperation between the authors. Judging from the results, Tian Jie is the author with the most publications and citations (n = 17, citation = 2128). The co-occurrence network map of cited authors is shown in Figure 6. Researchers have formed 2 major author cooperation groups with Fusco and Boldrini as the core. The cooperation within the groups is relatively close, and there is less cooperation between authors between groups. The cooperative ties between authors between groups should be strengthened. In addition, according to the calculation formula of the core author in Price law

$$\begin{array}{l} \begin{array}{l} M\;\approx 0.749\sqrt{Nmax}\; \end{array} \end{array}$$ (1)

Among them, *M* is the number of documents, and *N*max is the number of documents published by the author. The author with the most published documents is 17. According to Price law, the number of core documents is calculated to be about 3. Among all 2632 authors, 266 authors have more than 3 documents.

### 3.5. Analysis of publishing institutions and journals in the number of publications on CRC research using radiomics

By analyzing the publication status of different publishing institutions and journals, we can understand the application of radiomics technology in the field of CRC. Use VOSviewer to statistically analyze the publication volume of different publishing institutions and journals (Tables 1–2). The results show that the top 5 publishing institutions are GE Healthcare (32 documents), Fudan University (24 documents), Sun Yat-Sen University (22 documents), Sichuan University (19 documents), and Chinese Academy of Sciences (17 documents), except for GE Healthcare, they are all Chinese institutions. The institutions with more publications and greater influence are all distributed in China and the United States. The top 5 journals by the number of published documents are *Frontiers in Oncology* (60 documents), *Abdominal Radiology* (31 documents), *Cancers* (31 documents), *European Radiology* (28 documents), and *Academic Radiology* (14 documents).

**Table 1:** Top 10 institutions with the most publications on radiomics applied to CRC clinical research.

Rank	Organization	Country	Documents	Citations
1	GE Healthcare	the United States	32	392
2	Fudan University	China	24	391
3	Sun Yat-Sen University	China	22	355
4	Sichuan University	China	19	143
5	Chinese Academy of Sciences	China	17	1764
6	GE Healthcare China	China	17	339
7	University Chinese Academy of Sciences	China	16	760
8	Memorial Sloan Kettering Cancer Center	the United States	16	487
9	China Medical University	China	15	179
10	Southern Medical University	China	14	1546

CRC = colorectal cancer.

**Table 2:** Top 10 journals with the most published articles on the application of radiomics in CRC clinical research.

Rank	Journal	Country	Documents	Citations
1	*Frontiers in Oncology*	Switzerland	60	558
2	*Abdominal Radiology*	India	31	358
3	*Cancers*	Switzerland	31	435
4	*European Radiology*	Germany	28	776
5	*Academic Radiology*	the United States	14	225
6	*World Journal Of Gastroenterology*	China	13	133
7	*Radiology Medical*	Italian	11	461
8	*European Journal of Radiology*	Ireland	9	145
9	*Diagnostics*	Switzerland	8	92
10	*Scientific Reports*	the United Kingdom	8	256

CRC = colorectal cancer.

### 3.6. Analysis of cited literature related to the application of radiomics in CRC research

Citespace software was used to analyze cited documents related to the application of radiomics in CRC clinical research. The literature time slice was set to 1 year, and the node threshold parameter g-index was set to 10 according to the node situation. The most cited document is a document titled “Radiomics: Images Are More than Pictures, They Are Data” published by Gillies in 2016. The article details the process, challenges, and potential ability of radiomics to facilitate better clinical decision-making (Table 3).

**Table 3:** Top 10 cited references for radiomics application in CRC clinical studies.

Rank	Article	Citations	Year
1	Radiomics: images are more than pictures, they are data	97	2016
2	Radiomics analysis for evaluation of pathological complete response to neoadjuvant chemoradiotherapy in locally advanced rectal cancer	96	2017
3	MR imaging of rectal cancer: radiomics analysis to assess treatment response after neoadjuvant therapy	92	2018
4	Radiomics: the bridge between medical imaging and personalized medicine	92	2017
5	Computational radiomics system to decode the radiographic phenotype	72	2017
6	Radiomics analysis of multiparametric MRI for prediction of pathological complete response to neoadjuvant chemoradiotherapy in locally advanced rectal cancer	71	2018
7	Development and validation of a radiomics nomogram for preoperative prediction of lymph node metastasis in colorectal cancer	66	2016
8	Rectal cancer: assessment of neoadjuvant chemoradiation outcome based on radiomics of multiparametric MRI	61	2016
9	The image biomarker standardization initiative: standardized quantitative radiomics for high-throughput image-based phenotyping	48	2020
10	Can CT-based radiomics signature predict KRAS/NRAS/BRAF mutations in colorectal cancer?	46	2018

CRC = colorectal cancer, CT = Computed Tomography, MRI = magnetic resonance imaging.

### 3.7. Keyword co-occurrence analysis using radiomics in CRC research

**Figure 7.**
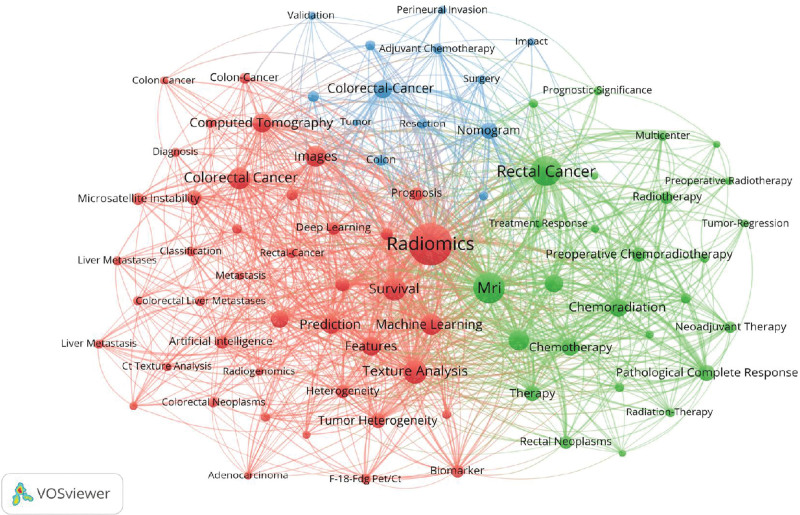
Radiomics applied to CRC clinical research keyword network analysis map. CRC = colorectal cancer.

Keywords are a high-level summary of the topic of the article, which can represent the core content of the article. Their frequency, relevance, and emergence can reveal the research hot spots, internal connections, and importance of the field. Use VOSviewer to draw the keyword co-occurrence network map and keyword heat map (Fig. 7), limiting the inclusion of studies where keywords appear more than 10 times, the results show that the number of nodes in the keyword co-occurrence network is 82, and the total number of connections is 16,826. The larger the node, the more times the keyword appears in the node. The keywords are sorted by frequency of occurrence. The top 10 keywords are Radiomics, Magnetic resonance imaging (MRI), Rectal cancer, Texture analysis, survival, Colorectal cancer, Images, Machine learning, Neoadjuvant chemoradiotherapy, and Features. These directions are the current hot spots in the clinical application of radiomics research in CRC and may be the direction of continued development in the future.

## 4. Discussion

Research on radiomics in the field of CRC has developed rapidly in recent years. The number of relevant publications and the frequency of citations has grown steadily. Imaging examinations provide abundant imaging data for artificial intelligence. Due to the explosive growth of imaging and clinical data, as well as breakthrough research on artificial intelligence, artificial intelligence represented by radiomics has shown great application potential in the clinical field of CRC, providing a new auxiliary way for clinicians to identify high-risk patients, select accurate and personalized treatment plans, and predict prognosis.^[11,12]^ Artificial intelligence represented by radiomics has played a very important role in CRC preoperative diagnosis, efficacy evaluation, prognosis prediction, and genotyping. From the perspective of research distribution, CRC radiomics research is mainly concentrated in China, the United States, Italy, and other countries. Among them, China has generated rich clinical data based on a large number of cases combined it with advanced computer technology, and contributed important practical application results. The studies on the application of radiomics in CRC that we included in the bibliometric analysis mainly involve the following topics.

### 4.1. Application of radiomics in preoperative diagnosis of CRC

Early-stage CRC can be directly surgically resected, while in a locally advanced stage, neoadjuvant chemoradiotherapy is advocated before surgery to downstage the tumor before surgery, improve tumor resectability, and reduce postoperative local recurrence. Therefore, accurate diagnosis and staging of CRC before surgery can help formulate the best individualized treatment plan for patients, and can also better assess the patient’s risk level. Grosu et al^[13]^ used machine learning methods to distinguish benign polyps and precancerous polyps in asymptomatic CRC screening patients and verified them using radiomics methods in an external validation set. The results showed that the area under the receiver operating characteristic curve (AUC) in the external validation set using radiomics methods was 0.91 (95% CI: 0.85–0.96) and the sensitivity was 82% (95% CI: 0.74–0.91), the specificity is 85% (95% CI: 0.72–0.95), and it can noninvasively distinguish between benign polyps and precancerous polyps before surgery. Sun et al^[14]^ retrospectively collected MRI data of 119 patients with rectal cancer, extracted 256 radiomics features on the T2WI sequence, and used cluster analysis and least absolute shrinkage and selection operator regression to predict the patient’s T stage. The AUC of this prediction model was 0.852 (95% CI: 0.67–1), the sensitivity was 79.0%, and the specificity was 82.0%. Wei et al^[15]^ collected magnetic resonance T2-weighted images (T2WI) and amide proton transfer-weighted images of 125 patients with rectal cancer and extracted radiomic features. Correlation analysis and multiparameter logistic regression analysis were used to select the most relevant radiomics features and clinical features, and clinical models, radiomics models, and clinical-radiomics combined models were constructed for preoperative prediction of lymph node metastasis of rectal cancer. The results showed that the clinical-radiomics combined model had the best effect in predicting lymph node metastasis of rectal cancer before surgery, and the AUC in the validation set was 0.929 (95% CI: 0.72–0.94). Wu et al^[16]^ extracted radiomics features from ultrasound images of 203 rectal cancer patients and established an ultrasound-based radiomics model to predict the lymphovascular invasion status of rectal cancer patients before surgery. The results show that the AUC of the training set of the ultrasound-based radiomics model is 0.849 and the AUC of the validation set is 0.781, which can better predict the lymphovascular invasion status of patients with rectal cancer before surgery.

### 4.2. Application of radiomics in CRC efficacy evaluation

Neoadjuvant treatment of rectal cancer has become a research hotspot at home and abroad. Preoperative neoadjuvant radiotherapy and neoadjuvant chemotherapy can significantly reduce the size of rectal cancer and achieve downstaging, thereby increasing the surgical resection rate and reducing the chance of local recurrence and metastasis.^[17–19]^ With the continuous development of radiomics, the application of radiomics in the evaluation of CRC efficacy is gradually increasing.^[20]^ Shi et al^[21]^ retrospectively collected T2-weighted Imaging (T2WI), diffusion weighted imaging, and dynamic contrast-enhanced sequences of patients with locally advanced rectal cancer (LARC) who received neoadjuvant chemoradiotherapy (nCRT), and extracted radiomics features from them to predict the pathological complete response of LARC patients after nCRT. The results showed that in the prospective validation set, the radiomics model AUC = 0.84 (95% CI: 0.70–0.94), and the radiomics model could effectively predict pathological complete response after nCRT. Petresc et al^[22]^ collected preoperative MRI images of 67 LARC patients who underwent nCRT and total mesorectal excision, and extracted radiomics features from the T2WI sequences to predict the treatment response of LARC patients to nCRT. The results show that the T2WI radiomics model can predict the treatment response of LARC patients to nCRT before surgery, with the training set AUC = 0.94 (95% CI: 0.82–0.99) and the validation set AUC = 0.80 (95% CI: 0.58–0.94). Zhou et al^[23]^ used deep neural network to extract Positron Emission Tomography/Computed Tomography (PET/CT) image features, clinical data, and histological protein biomarkers from PET/CT images to construct a multimodal model for predicting the response of colorectal liver metastases to bevacizumab. The results showed that the multimodal model accurately predicted the therapeutic response to bevacizumab in patients with colorectal liver metastases, with an external validation set AUC = 0.83 (95% CI: 0.75–0.92), a sensitivity of 80.4%, and a specificity of 76.8%. Huang et al^[24]^ found that integrating a radiomics model based on CT images into RNA immunogenomic expression data can effectively predict cancer recurrence. Correlation between radiomic signatures and immunogenomic expression may also provide biological relevance and personalized therapeutic targets.

### 4.3. Application of radiomics in CRC prognosis prediction

Nowadays, advances in surgery and nCRT have significantly improved the outcomes of CRC patients, but postoperative local recurrence and metastasis are still key negative prognostic factors.^[25]^ Radiomics has also made certain progress in the prognosis of CRC. Kong et al^[26]^ studied the non-contrast-enhanced and contrast-enhanced CT images of 84 CRC patients undergoing surgical treatment and extracted radiomics features from them to establish an image-clinical combined omics model for predicting the prognosis of postoperative CRC patients. The results show that the imaging-clinical combined omics model can predict the overall survival (OS) of patients, with a C-index of 0.821 (95% CI: 0.74–0.89). Wang et al^[27]^ studied 18F-FDG PET/CT images of 124 patients with stages III-IV CRC who received treatment, used least absolute shrinkage and selection operator Cox regression to select radiomic features, and constructed a radiomics model was developed to predict the prognosis of CRC patients. The results show that the PET/CT radiomics model can better predict the progression-free survival and OS of CRC patients. The C-index of the radiomics model is 0.712 (95% CI: 0.68–0.74) and 0.758 (95% CI: 0.72–0.78). Lee et al^[28]^ also studied the application of 18F-FDG PET/CT images in predicting the prognosis of 91 patients with stage IV CRC under treatment, and the results showed that the PET/CT radiomics model could effectively predict the OS of patients with stage IV CRC, HR = 4.498 (95% CI: 1.024–19.759). Zhu et al^[29]^ and Li et al^[30]^ constructed a radiomics model based on CT images that can be used to predict the prognosis of CRC patients. Yang et al^[31]^ also constructed a radiomics model to predict the prognosis of CRC patients based on MRI images. The results showed that the MRI-based radiomics model can also better predict the prognosis of CRC patients and provide risk stratification. Dohan et al^[32]^ developed a delta radiomics model that can effectively predict the OS of metastatic CRC patients who received FOLFIRI and bevacizumab as first-line treatment. Multimodal radiomics plays an increasingly important role in predicting the prognosis of CRC patients.

### 4.4. Application of radiomics in CRC genotyping

Genomic analysis is critical for the treatment of CRC. Genomic analysis can help identify specific genetic mutations that drive the growth of a patient’s cancer, and this information can be used to guide treatment decisions.^[33,34]^ Radiomics can noninvasively predict CRC gene expression before surgery and provide personalized treatment decisions for CRC patients. Chen et al^[35]^ extracted radiomics features from preoperative enhanced CT images of CRC patients and used the artificial neural network model enhanced by genetic algorithm to screen out microsatellite instability (MSI) related radiomics features to establish a radiomics model to predict the MSI status of CRC patients. The results showed that the radiomics feature model showed robust prediction performance in both internal and external validation cohorts, with AUCs of 0.788 and 0.775, respectively, and could better predict the MSI status of CRC patients before surgery. Zhong et al^[36]^ collected MRI images of 300 patients undergoing radical resection of rectal cancer extracted radiomics features from T2WI sequences and combined them with clinical data to establish a radiomics nomogram for preoperative prediction of p53 gene mutation status in rectal cancer patients. The results showed that the radiomics nomogram can effectively predict the mutation status of the p53 gene, and the AUC of the training set and validation set were 0.828 and 0.795 respectively. Porto-Álvarez et al^[37]^ and Hu et al^[38]^ found that a radiomics model based on CT images can also be used to predict the KRAS gene mutation status in CRC patients before surgery. Jing et al^[39]^ built a radiomics model based on MRI that can be used to predict the rectal mismatch repair status of CRC patients before surgery. At the same time, Cao et al^[40]^ also built a model based on CT images. The results of the radiomics model show that it can also better predict the mismatch repair status of CRC patients before surgery. Multimodal radiomics models can noninvasively predict the mutation status of multiple genes before surgery and can provide patients with more personalized treatment decisions before surgery.

### 4.5. Challenges and future developments of radiomics

Medical imaging data has been used for a long time in some fields such as radiology, but the size of these data sets is still relatively small, which limits the development of imaging omics to some extent.^[41]^ Most current radiomics models are limited to images from a single medical institution, which may lead to model overfitting and models that are not fully clinically applicable. Therefore, in the construction of radiomics models, external validation is necessary^[42]^ to improve the generalization ability and prediction accuracy of the model. Due to the relatively short development time of radiomics technology, unified standards, and specifications have not yet been formed, which makes it difficult to share and compare research results between different research institutions. In recent years, with the continuous deepening of radiomics research, some public imaging databases such as TCIA, MedPix, LONI, etc, have also been gradually established and improved. The establishment of these databases provides a large amount of data support for radiomics research and promotes the development of radiomics.^[43]^ At the same time, we should also be aware that in the process of establishing a public medical imaging database, many technical and management problems need to be solved, such as data collection, organization, storage, and sharing. Therefore, we need to strengthen interdisciplinary cooperation and exchanges, improve data quality and standardization, and strengthen technology research and development and transformation to promote the further development of medical imaging public databases. In multicenter experiments in radiomics, image data, and source data need to be shared to create a database large enough for statistical analysis. However, data sharing often encounters cultural, administrative, regulatory, and other issues. At the same time, since medical imaging data involves patients’ private information,^[44]^ how to protect patients’ privacy while sharing data and avoiding abuse and infringement is an urgent problem to be solved.^[45]^ With the development of radiomics technology, more and more models are used in the analysis of medical images. However, these models often have problems such as large amounts of calculation and slow running speed, which to a certain extent affects their promotion in practical applications.^[46]^ In addition, although the performance of the model is excellent, it often lacks interpretability,^[47]^ which makes it difficult for doctors to understand the decision-making process of the model, thus raising doubts about its application in clinical practice. Therefore, we may need more interpretable models in the future, such as linear models or decision tree models for clinical problem research.^[47]^ Although radiomics technology has broad application prospects in the medical field, most current research results are still in the laboratory stage and have not yet achieved large-scale clinical application.^[48]^ This is mainly due to the influence of various factors such as technology maturity, equipment costs, policies, and regulations. Therefore, how to transform radiomics technology into actual clinical applications is an important challenge currently faced.

## 5. Limitations

As English is the most commonly used and widely disseminated language in the field of scientific research, second, the acquisition and retrieval of English literature is relatively more convenient and efficient. Due to the limitations of bibliometric analysis software and visualization software, this study only included English literature in the WoSCC database, which would lead to selection bias. To obtain more comprehensive and accurate research results, we will strive to overcome language barriers and expand the scope of literature sources as much as possible in the future. Due to the large number of retrieved documents, we only conducted an in-depth analysis of the top-ranked institutions, authors, and journals, and were unable to analyze specific documents one by one. In academic research, due to various reasons, publication bias may occur in research results. In the future, we will adopt the methods of expanding the scope of literature retrieval and evaluation using imputation to minimize the impact of publication bias on the results as much as possible. The articles we included are current as of October 31, 2023. However, the database is still being updated and we were unable to include the latest publications.

## 6. Conclusions

Through bibliometric and visual analysis of scientific results and research trends of radiomics in CRC research. Radiomics has shown great application potential in various clinical aspects of CRC, such as preoperative diagnosis, efficacy evaluation, prognosis prediction, and genotyping of CRC patients. Radiomics promises to have a practice-changing impact on the clinical field of CRC. Clinicians can reexamine and adjust their diagnostic and treatment strategies based on the latest findings of CRC radiomics research, for instance, by combining radiomics features for precise diagnosis and developing individualized treatment plans. Researchers can further explore areas that have not been fully studied and adopt more advanced technical methods to improve the accuracy of research, such as introducing artificial intelligence algorithms like machine learning and deep learning and strengthening interdisciplinary collaboration between medical and engineering disciplines. Administrators can adjust relevant medical policies based on the progress of CRC radiomics research, such as establishing radiomics data-sharing platforms and developing application standards for radiomics technology. However, we must admit that there are still many shortcomings and challenges in radiomics, such as single-source imaging data,^[49]^ privacy protection for medical data sharing,^[50]^ model interpretability,^[51]^ and clinical translation of radiomics technology. Overall, radiomics has shown great potential in the clinical application of CRC, but many challenges still need to be solved to achieve its widespread application. Through research on cross-source data integration, privacy protection, data sharing, development of interpretable models, and clinical translation, we can expect radiomics to play a greater role in the clinical practice of CRC.

## Author contributions

Data curation: Lihong Yang, Xiaoying Shi, Bairu Li, Jiaqiang Xie.

Methodology: Lihong Yang.

Software: Lihong Yang.

Writing—original draft: Lihong Yang.

Writing—review & editing: Binjie Wang, Changfu Wang.

Funding acquisition: Changfu Wang.
